# Outcome of antidepressant drug discontinuation with taperingstrips after 1–5 years

**DOI:** 10.1177/2045125320954609

**Published:** 2020-09-02

**Authors:** Peter C. Groot, Jim van Os

**Affiliations:** Department of Psychiatry, University Medical Centre Utrecht, Postbus 85500, Utrecht, 3508 GA, The Netherlands; Department of Psychiatry and Neuropsychology, Maastricht University Medical Centre, Maastricht, The Netherlands; Department of Psychosis Studies, King’s College London, King’s Health Partners, Institute of Psychiatry, London, UK; User Research Centre Netherlands, UMC Utrecht Brain Centre, University Medical Centre Utrecht, Utrecht, The Netherlands

**Keywords:** antidepressants, dependence, drug withdrawal symptoms, patient medication knowledge, tapering

## Abstract

**Background:**

Stopping antidepressants is often difficult due to withdrawal. Taperingstrips were developed to facilitate antidepressant discontinuation according to the recently described Horowitz-Taylor method, allowing for personalised titration of discontinuation to the intensity of withdrawal. A taperingstrip consists of antidepressant or other medication, packaged in a 28-day roll of small daily pouches, each with the same or slightly lower dose than the one before it. We previously reported that the short-term success rate of antidepressant taperingstrips was 71%. Here, we examine longer-term outcome after 1–5 years.

**Methods:**

Patients whose doctor had ordered taperingstrips between January 2015 and December 2019 were sent a questionnaire for participation in anonymised research in January 2020. Of 1012, 483 participated, of whom 408 (85%) had attempted antidepressant tapering.

**Results:**

Of the 408 patients included, 192 (47%) had used strips for tapering venlafaxine, 142 (35%) for paroxetine and 74 (18%) for other antidepressants. Median length of antidepressant use was 4 years, and most (61%) had tried to come off without taperingstrips at least once. After 1–5 years, 270 patients (66%) remained off antidepressants after tapering their antidepressant, 6 (2%) had successfully reduced their medication, 87 (21%) had restarted due to (self-reported) relapse, 35 had restarted for another indication (9%), and 10 (3%) reported another outcome. People with more severe experience of withdrawal prior to tapering, and people who had been on antidepressants for a shorter period of time, were more likely to remain off medication after 1–5 years.

**Conclusion:**

The previously reported 71% short-term success rate of taperingstrips in the most severely affected group, was matched by a 68% rate after 1–5 years. The evidence-based approach of personal tapering to counter withdrawal, as used for drugs causing withdrawal, for example, benzodiazepines, may represent a simple solution for an important antidepressant-related public health problem, without extra costs.

## Introduction

It is widely recognised that a significant proportion of users of modern selective serotonin reuptake inhibitor (SSRI) and serotonin and norepinephrine reuptake inhibitor (SNRI) antidepressants experience difficulties coming off medication,^1,2^ although differences between studies make it difficult to give a precise summary estimate of incidence. Withdrawal effects threaten the internal (and external) validity of antidepressant maintenance trials due to withdrawal confounding.^3^ In addition, given high rates of chronic prescribing, particularly in deprived areas,^4^ withdrawal and inability to discontinue antidepressant medication represent a significant public health problem. There is debate as to what degree withdrawal, in the context of antidepressant cessation, can be taken to reflect dependence.^5,6^ Practically, however, viewing antidepressant withdrawal through the prism of withdrawal of other drugs, such as benzodiazepines, has much to offer given the solid evidence base supporting treatment of withdrawal by gradual and personal tapering of the substance in question, titrated against the degree of discomfort caused by withdrawal. The recently described Horowitz-Taylor method of withdrawal represents such a common sense strategy.^7^ It remains difficult to implement for antidepressants, however, given that virtually all medications come in dosages that do not allow for flexible and personal tapering.^8^ The Horowitz-Taylor method recognises that antidepressant tapering should be ‘hyperbolic’, meaning the dose needs to be reduced by half, then by half of that, and so on.^7^ Although some antidepressants can be cut or come in fluid form, these methods are cumbersome and imprecise,^9^ and not developed specifically for tapering.

In recent years, in response to users of antidepressant medication trying to come off, the not-for-profit organisation Cinderella Therapeutics in the Netherlands oversaw the development of personal taperingstrips for hyperbolic reduction of antidepressant medication in those suffering withdrawal or deemed at risk.^8,10^ A taperingstrip consists of antidepressant medication, packaged in a roll or strip of small daily pouches. Each pouch is numbered and has the same, or slightly lower, dose than the one before it. Strips come in series covering 28 days, and patients can use one or more strips to regulate the rate of dose reduction over time. Dose and day information printed on each pouch allow patients to precisely record and monitor the progress of their reduction.^8,10^

In a previous study, we reported that 71% of patients using a taperingstrip, mainly for venlafaxine and paroxetine, were able to come off their drug in the short term, reporting the outcome after a 2–3 month course with taperingstrips, using a median of two 28-day taperingstrips.^11^ Here, we report on the longer term, 1–5 year outcome of antidepressant taperingstrips in a sample of 483 users of psychotropic drugs in the Netherlands, of whom 408 used an antidepressant. The main outcome was the proportion of users of taperingstrips who indicated they were off antidepressant drugs after a minimum period of 1 year. In addition, guided by previous work,^1,2,11^ we tested for association with hypothesized predictors including length of antidepressant use, number of previous attempts with taperingstrips, level of previous withdrawal in those with previous attempts, and demographic factors.

## Methods

Participants were patients whose doctors had ordered taperingstrips from the pharmacy manufacturing the strips over the period 1 January 2015–31 December 2018. Patients responded to a routine and anonymous quality assessment questionnaire which was sent in January 2020 (483/1012, response rate 48%), meaning patients were assessed 1–5 years after tapering their medication with a minimum of one full year. Only individuals who had attempted tapering of a named antidepressant were included in the analysis (*n* = 408, 85%). Under Dutch law, medical ethical approval is not required for analysis of anonymous routine quality assessment data. Patients were given information about the purpose of the questionnaire and were informed that, if they wished to consent to participation in anonymized research, they could send back a completed questionnaire. No identifiers were collected and no reminders were sent.

Some of the responders may have participated in our previous investigation reporting on the short-term outcomes of tapering strips, based on a different questionnaire that patients used to report on outcome in the short term after a 2–3 month period of using taperingstrips to come off antidepressant medication.^11^

Variables collected were: (i) age (in years); (ii) sex; (iii) type of medication and length of use prior to tapering; (iv) number of attempts to stop medication before using taperingstrips; (v) number of withdrawal symptoms during previous attempts out of a list of eight symptom groups, as described by Groot,^12^ and included in the recent Dutch consensus document on tapering (Table 1)^13^; (vi) number of withdrawal symptoms experienced with taperingstrips out of the same list of eight symptom groups (Table 1); (vii) outcome of tapering after 1–5 years (still off medication; on medication but lower dose; tapered but restarted medication due to relapse of illness; tapered but restarted medication for another indication; other). An additional dichotomous outcome was used in the analyses, comparing those still off medication (*n* = 270, 66% of total), or using a lower dose (*n* = 6, 2% of total), with the other three outcome categories (*n* = 132, 32%). This dichotomous outcome (*n* = 276, 68%) will hereafter be referred to as ‘dichotomous success’.

**Table 1.** Eight groups of withdrawal symptoms of SSRIs and SNRIs.

- Flu-like symptoms; such as headache, lethargy, sweating, chills, tiredness, loss of appetite, muscle pain - Sleep disorders; such as poor sleep and nightmares - Gastrointestinal symptoms; such as nausea, vomiting, diarrhoea and anorexia - Balance problems; such as dizziness and coordination disorders - Sensory symptoms; such as sensations of electrical shocks, paraesthesias and palinopsia (prolonged after-images) - Mental complaints; such as anxiety, gloominess and irritability/irritation or the occurrence of (hypo-) mania (disinhibition) - Extrapyramidal symptoms; such as movement disorders and tremors - Other symptoms; such as cognitive alterations and cardiac arrhythmias.

SSRI, selective serotonin reuptake inhibitor; SNRI, serotonin and norepinephrine reuptake inhibitor.

Taperingstrips were prescribed for a wide variety of mainly antidepressant medications (Table 2). In the analyses, these were reduced to three groups: venlafaxine (*n* = 192, 47%), paroxetine (*n* = 142, 35%) and other antidepressant (*n* = 74, 18%).

**Table 2.** Antidepressant medications for which taperingstrips were prescribed.

Medication	Frequency	Percentage
Amitriptyline	7	1.7
Bupropion	5	1.2
Citalopram	19	4.7
Duloxetine	1	0.3
Escitalopram	3	0.7
Fluoxetine	8	2.0
Fluvoxamine	3	0.7
Mirtazapine	14	3.4
Paroxetine	142	34.8
Sertraline	14	3.4
Venlafaxine	192	47.1
Total	408	100

Multivariable logistic regression was conducted to examine associations between dichotomous success after 1–5 years after tapering the antidepressant medication as dependent variable and other variables as independent variables. As there were no large or significant interactions with the three categories of antidepressant medication for any variable, results are shown for all antidepressant medications combined. As ratings of previous withdrawal applied only to the subgroup of individuals with previous attempts at discontinuation (*n* = 250, 61%), logistic regression models were run twice: one using the full sample without the variable indexing previous withdrawal, and one in the subgroup with previous attempts including the variable indexing previous withdrawal. Associations were checked for consistency across the two models.

## Results

Out of 1012 individuals who were sent a questionnaire, 483 returned a completed questionnaire, of whom 408 had attempted to taper a named antidepressant. The majority was female (70%), and mean age was 52.0 years [standard deviation (SD) = 14.5]. Median number of years of use of medication was 4, during which most had had previous attempts to stop without taperingstrips (61%), during which they had experienced a median of three withdrawal symptoms, compared with a median of one withdrawal symptom using taperingstrips in the entire sample (Table 3).

**Table 3.** Data from returned questionnaires.

Medication		Age	Years of use	Previous attempts	Previous withdrawal*	Current withdrawal**		Female sex	First attempters^‡^	Dichotomous success^†^
Venlafaxine (*n* = 192)	Mean	52.0	6.4	1.0	3.6	1.2	%	72.3%	37.0%	68.8%
	SD	14.6	5.8	1.1	1.8	1.5				
	Median	54	5	1	3	1				
	Min	18	0	0	1	0				
	Max	85	25	4	8	8				
Paroxetine (*n* = 142)	Mean	53.7	7.6	1.2	3.3	1.1	%	63.6%	39.4%	64.8%
	SD	13.5	6.9	1.2	1.8	1.6				
	Median	56	5	1	3	0				
	Min	21	0	0	0	0				
	Max	80	25	4	8	8				
Other antidepressant (*n* = 74)	Mean	48.8	6.3	1.0	3.2	1.0	%	77.0%	41.9%	70.3%
	SD	15.7	7.2	1.2	2.0	1.5				
	Median	49	3	1	3	0				
	Min	19	0	0	0	0				
	Max	82	35	4	8	8				
Total (*n* = 408)	Mean	52.0	6.8	1.1	3.4	1.1	%	70.1%	38.7%	67.6%
	SD	14.5	6.5	1.1	1.8	1.5				
	Median	54	4	1	3	1				
	min	18	0	0	0	0				
	max	85	35	4	8	8				

* Withdrawal experienced in the group with previous attempts before using taperingstrips; withdrawal defined as number of withdrawal symptoms out of a group of eight symptoms.

** Withdrawal experienced during the use of taperingstrips.

† Defined as still off medication (*n* = 270, 66%) or using a lower dose (*n* = 6, 2%).

‡ Subgroup who never attempted to come off medications before.

*n*, total number of individuals; SD, standard deviation.

The distribution of the outcomes after 1–5 years were: still off medication: *n* = 270 (66%); on medication but using a lower dose: *n* = 6 (2%); tapered but restarted medication due to (self-assigned) relapse of illness: *n* = 87 (21%); tapered but restarted medication for another indication: *n* = 35 (9%); other: 10 (3%). Dichotomous success rate combining the first two categories as ‘success’ was *n* = 276 (68%). The three antidepressant medication groups did not show large differences in these mean/median and proportions.

Dichotomous success was distributed approximately equally across age, sex, number of previous attempts and proportion of first attempters, and taperingstrip withdrawal (Table 4). In those who were still successfully off medication after 1–5 years, number of years of use of medication prior to tapering appeared to be lower, and number of withdrawal symptoms during previous attempts appeared to be higher (Table 4).

**Table 4.** Distribution of successful tapering, 1–5 years after coming off the antidepressant.

Success^†^		Age	Years of use	Previous attempts	Previous withdrawal*	Current withdrawal**	Female sex	First attempters^‡^
No success (*n* = 132)	Mean	53.7	9.0	1.2	3.1	1.3	73.3%	37.1%
	SD	13.4	7.4	1.3	1.7	1.5		
	Median	56	7	1	3	1		
	Min	21	0	0	0	0		
	Max	78	35	4	7	8		
Success (*n* = 276)	Mean	51.2	5.8	1.0	3.6	1.0	68.6%	39.5%
	SD	15.0	5.7	1.1	1.9	1.5		
	Median	54	3	1	3	0		
	Min	18	0	0	0	0		
	Max	85	25	4	8	8		

* Withdrawal experienced in the group with previous attempts before using taperingstrips; withdrawal defined as number of withdrawal symptoms out of a group of eight symptoms.

** Withdrawal experienced during the use of taperingstrips.

† Defined as still off medication (*n* = 270, 66%) or using a lower dose (*n* = 6, 2%).

‡ Subgroup who never attempted to come off medications before.

*n*, total number of individuals; SD, standard deviation.

Logistic regression confirmed these descriptives. In the model including age, sex, medication group, years of use, number of previous attempts and number of withdrawal symptoms during tapering, more years of use prior to tapering was associated with a lower probability of successful tapering after 1–5 years [odds ratio (OR) = 0.93, 95% confidence interval (CI): 0.90, 0.97, *p* = 0.001]. No large or significant associations were apparent for the other variables in the model (age: OR = 1.00, *p* = 0.605; sex: OR = 0.85, *p* = 0.537; paroxetine (*versus* reference venlafaxine): 0.80, *p* = 0.384; other medications (*versus* reference venlafaxine): 0.96, *p* = 0.893; number of previous attempts: OR = 1.01, *p* = 0.932; number of withdrawal symptoms with taperingstrips: OR = 0.95, *p* = 0.513). In the same model, but additionally including number of withdrawal symptoms during previous attempts, as explained in the analysis section, a greater number of withdrawal symptoms during previous attempts predicted dichotomous success 1–5 years after tapering the antidepressant medication (OR = 1.30, 95% CI: 1.06, 1.60, *p* = 0.012). In this model, more years of use prior to tapering also predicted lower probability of dichotomous success (OR = 0.91, 95% CI: 0.87, 0.97, *p* = 0.001), but not any of the other variables, consistent with the model with the full sample.

## Discussion

### Findings

The results of the current study show that the high short-term rate of successfully coming off antidepressant medications (71%) is matched by an equally high success rate at the longer term 1–5 years after tapering the antidepressant medication (68%).^11^ As patients necessitating prescription of a taperingstrip can be considered as the group most severely affected – as evidenced by the high rate of previous attempts, long period of use of antidepressant medication and the fact that their doctor had decided to prescribe a taperingstrip – the results also serve as an independent, longer-term replication of the fact that tapering has a high success rate in those at the highest end of the severity spectrum of antidepressant withdrawal.

The fact that the cost of a 28-day taperingstrip of venlafaxine in the Netherlands is not far from the yearly price of taking venlafaxine may point to cost-effectiveness. In the Netherlands, 4 years of venlafaxine use (the median length of use in this sample) of 37.5 mg b.d. would cost around 380 euros, whereas a 28-day course with a custom-made taperingstrip would cost around 150 euros. This comparison, however, only considers direct costs and ignores the somatic and mental burden of unnecessary use of psychotropic medications and the impact on quality of life.

The results indicated that the higher the number of years of use, the greater the difficulty coming off medications. Longer-term use of antidepressants is a recognised risk factor for withdrawal – a worrying statistic given high rates of chronic use of antidepressants, particularly in deprived areas.^4,14,15^ The finding is validated by our previous report, in which we found that patients who had used antidepressants for longer periods of time required more extended tapering to come off.^11^ The mechanisms for the relationship between longer use and more difficulties tapering antidepressants are not known. Even short-term use of antidepressants induces a range of brain and metabolic changes,^16^ and there is meta-analytic evidence suggesting that chronic use of medication may make patients more relapse-prone.^17^

Our study also suggests that those with previous experience of more severe withdrawal are more likely to benefit from taperingstrips. This findings replicates the results of our previous study on the short-term outcome of tapering, in which previous experience of withdrawal also was less in those who did not succeed in coming off their antidepressant medication after a 2–3 month period of tapering.^11^ On the one hand, this may be considered an encouraging finding, as it suggests that tapering can provide a workable solution for those with the severest manifestation of withdrawal. This finding is validated by the substantial difference in reported withdrawal between attempts at discontinuation without taperingstrips (median number of withdrawal symptoms = 3) and with taperingstrips (median number of withdrawal symptoms = 1). The (replicated) finding that more severe previous withdrawal predicts successful tapering may, on the other hand, be related to several important clinical factors. For example, persons with less previous withdrawal may be more likely to taper more rapidly (and persons with more severe experience of previous withdrawal more slowly), which in turn may cause withdrawal symptoms or relapse, given the fact that gradual tapering, in comparison with more abrupt discontinuation, is a protective factor against early relapse.^18,19^ Another explanation is that persons with less previous withdrawal, at a certain level of withdrawal in the initial stages of tapering, may be more likely to prematurely conclude (or their doctor concludes) that they need their medication and cannot stop. Finally, it may be possible that persons with more previous withdrawal are more motivated to stay off their medication after having tapered them successfully.

Although 68% remained off medication 1–5 years after using taperingstrips, nearly one-third of the sample had not been successful. As described elsewhere, rate of tapering can be a very important factor and some individuals may be able to come off medications if a slower and more personalised approach is adopted.^8^ In addition, shared decision making is crucial, as is following the principles of the Horowitz-Taylor method of hyperbolic tapering. Optimising these may help persons come off antidepressants even if previous tapering was not successful. Self-monitoring can sometimes add an extra dimension, making the tapering more personalised and more robust to manage early withdrawal.^8,20^ The most effective way to tackle the problem, however, is prevention, using psychotropic medications more conservatively and taking into account the fact that some compounds likely are more associated with withdrawal than others.

### Methodological issues

The results should be interpreted in the light of the following methodological issues. The sample was largely selected by the referring physician for unsuccessful previous withdrawal and chronic use of antidepressant medications. Although around 40% were first-attempters, these patients may represent a subgroup with risk factors in addition to chronic use, for example experiencing withdrawal symptoms when forgetting to take the medication for a few days (unfortunately this was not asked in the survey). In other words, the 68% rate of persistent success of taperingstrips 1–5 years after coming off the antidepressant, applies to the selected group considered most at risk of severe withdrawal and difficulties coming off antidepressant and other psychotropic medications. The external validity of the research therefore may extend to the group of people most at risk of withdrawal in countries with comparable rates of antidepressant prescription.

Around half the sample responded, with the possibility of bias due to selective non-response. However, even if the non-responder success rate had only been half that of the observed success rate, the overall rate in the entire sample would still be around 50%, which may still be considered a high percentage for the group most at risk of severe withdrawal and previous unsuccessful attempts at discontinuation. Nevertheless, further research is necessary for more precise quantification of persistent success following the use of taperingstrips.

In order to increase the response rate, the quality assurance questionnaire was kept brief, preventing us from more precise assessment of variables, for example, precise length of follow up after tapering the medication, nature and type of relapse, relationship with and support by prescriber, number of taperingstrips used, length of tapering, current level of symptoms, etc.

Patients self-reported ‘relapse’ and starting antidepressants again because of it. It is of course possible that a number of these relapses in fact concerned withdrawal syndromes that were interpreted as illness relapse.

Ideally, we would have been able to identify persons who had participated in our previous investigation on the immediate outcome of tapering. This would have enabled us to examine aspects of the early process of tapering in relation to the longer-term outcome after 1–5 years. There is no reason to assume, however, that earlier participation in a different, short-term investigation would bias the outcome of the longer-term endeavour reported here.

## Conclusion

Treating antidepressant and other medication withdrawal using the evidence-based strategy of gradual personal tapering, in accordance with the Horowitz-Taylor method,^7^ and titrated against the level of withdrawal experience, as used for drugs causing withdrawal such as benzodiazepines, appears to work for the majority of patients with chronic use and previous experience of withdrawal.
